# Oral *Candida albicans* colonization in healthy individuals: prevalence, genotypic diversity, stability along time and transmissibility

**DOI:** 10.1080/20002297.2020.1820292

**Published:** 2020-09-14

**Authors:** Ângela Gerós-Mesquita, Joana Carvalho-Pereira, Ricardo Franco-Duarte, Armandino Alves, Hernâni Gerós, Célia Pais, Paula Sampaio

**Affiliations:** a CBMA (Centre of Molecular and Environmental Biology) / Department of Biology / University of Minho, Braga, Portugal; b Centro De Ortodontia De Braga, Braga, Portugal; c CITAB (Centre for the Research and Technology of Agro-Environmental and Biological Sciences) / University of Trás-os-Montes and Alto Douro, Vila Real, Portugal; d CEB (Centre of Biological Engineering) /Department of Biological Engineering / University of Minho, Braga, Portugal

**Keywords:** Oral colonization, healthy individuals, yeasts, smoking habits, *Candida albicans*, cai genotype

## Abstract

In this study, 181 healthy individuals, including 29 couples, were analysed regarding oral yeast colonization using a culture-based approach. Results showed that 39% of the individuals were yeast carriers, 89% being colonized with *Candida albicans*, 5% with *C. guilliermondi*, 3% with *C. lusitaniae* and 3% with *C. parapsilosis*.

Sixty-two percent of the couples had at least one member colonized. Colonization and CFU counts were higher in the couples´ group. Eighty percent of the volunteers were colonized with *C. albicans* strains with only one CAI genotype, while two but similar CAI genotypes inhabited the oral cavity of the remaining 20% individuals. The same CAI genotypes were found in 66.6% of the couples when both were colonized.

Our results indicate that the intimacy among couples increases the probability of heavy cross-colonization, which is potentiated when one member of the couple is a smoker.

## Introduction

Advances in metagenomics allowed a more complete understanding of the different microorganisms that inhabit on or within human tissues and fluids. The number and type of these microbes vary with age, diet and personal hygiene habits, being collectively referred to as the normal microbiota of the human body. In particular, the oral cavity is colonized by a highly diverse set of microbes from different species, including bacteria, protozoa and fungi, and also by viruses [1,2]. They are always present in metabolic inter-dependent and highly organized polymicrobial communities on the surface of teeth, gums and tongue. The bacterial microbiome makes up over 99% of the total microbial counts and is coined as the core microbiome, while the remaining, less abundant and more diverse microbiota forms the ‘rare biosphere’ [3]. The mycobiome represents a significant proportion of the rare biosphere. In culture-independent studies, 85 fungal genera were reported in healthy hosts, with *Candida, Cladosporium, Aureobasidium, Saccharomycetales, Aspergillus, Fusarium* and *Cryptococcus* being most predominant [4]. In culture-dependent studies from salivary samples, the most predominant moulds were *Penicillium* spp., *Aspergillus* spp. and *Cladosporium*, while the most abundant yeasts were *Candida* spp. and *Rhodotorula* spp [5]. In these studies, *Candida* species were the most prevalent and existed commensally in the oral cavity of up to 70% of healthy individuals [6,7]. In fact, *Candida* species like *C. albicans, C. dubliniensis, C. parapsilosis* and *C. glabrata* belong to the normal microbiota of the skin and mucosal surfaces of healthy individuals, not only in the oral cavity, but also in the gastrointestinal tract and the vagina [8].

Despite its relatively low abundance, the impact of the mycobiome in human health and disease is well established [9, Nguyen 2015]. Reduced host defence or inadequate clearance promotes *Candida* opportunistic infection, leading to a spectrum of oral mucosal disorders, from simple to chronic candidosis [10]. In severely immunocompromised patients, such as those under chemotherapy, patients with AIDS or with endocrinal or blood diseases, the commensal fungi can proliferate, penetrate the bloodstream and disseminate throughout the human body causing life-threatening infections [8,11,12].

The increasing number of infections caused by *Candida* spp. and the appearance of strains resistant to conventional treatment, led to the development of new approaches to effectively identify different *Candida* spp., among which microsatellite typing was used [13,14]. The *C. albicans* microsatellite CAI was first described in 2003 and has proved to be an efficient method for strain differentiation with a discriminatory power of 0.97 [13].

Most studies on the prevalence of *C. albicans* in the human oral cavity have been performed in individuals with different pathologies or belonging to risk groups. It is widely described that *C. albicans* predominates among *Candida* spp. in the oral cavity, but little is known about its genotypic diversity, stability and transmissibility between individuals. Hence, the two main goals of the present study were to determine yeast colonization in the oral cavity of healthy individuals and to evaluate patterns of *C. albicans* transmission between couples, and their stability over time. Moreover, correlations with gender and smoking habits were also studied.

## Material and methods

### Participants and sampling

One hundred and eighty-one healthy individuals volunteered to participate in this study, including students and staff of the University of Minho. The study had been approved by the Ethics Committee for Research in the Life and Health Sciences of the University. All the participants signed an informed consent, and data regarding food habits, oral hygiene and smoking habits were registered. The ages ranged from 18 to 60 years, with 116 females and 65 males. Exclusion criteria included antibiotic or steroid therapy within the 3 months preceding the collection. To maintain the anonymity of the donors a number was assigned to each. Members of a couple received the same number, being distinguished only by the use of the letters ‘a’ (female) and ‘b’ (male). To assess individual variation of oral yeast colonization over time, a follow-up of 10 participants was carried out approximately 1 year after the first sampling. The selection criteria for the follow-up participants included identification of *C. albicans* in the first sampling and continued participant availability.

A saliva sample (approximately 1 mL) from each participant was collected between 10 a.m. and 12 a.m., to avoid variation in salivary flow rates, and directly plated and spread on Yeast extract glucose chloramphenicol agar (YGC) with 50 mg/L chloramphenicol to inhibit bacterial growth. The plates were incubated at 37°C for 5 days. The cultures were examined every day, and the number of fungal colonies was recorded in colony-forming units per millilitre (CFU/mL).

### Phenotypic and molecular identification of the isolates

The yeast colonies (a maximum of 50 per individual) were sub-cultured on CHROMagar Candida to facilitate the presumptive identification of *Candida* yeast species. Following this preliminary phenotypic characterization, isolates identified on the CHROMagar as *C. albicans* and the non-*albicans* species were further analysed through the use of molecular techniques. From each individual, a maximum of eight *C. albicans* colonies were randomly selected for CAI analysis, and one non-*albicans* colony of each different colour was chosen for ITS species identification. Bacteria isolates were not identified in this study.

### DNA extraction, amplification, and sequencing conditions

Before DNA isolation, cells were cultivated overnight on Sabouraud medium at 30°C and DNA was extracted according to [15]. Isolates presumptively identified as *C. albicans* were genotyped with the CAI species-specific microsatellite marker. CAI locus was amplified with primers 5´-ATG CCA TTG AGT GGA ATT GG-3´ (forward) and 5´-AGT GGC TTG TGT TGG GTT TT-3´ (reverse), according to [13]. PCR products were analysed by capillary electrophoresis in an ABI 310 genetic analyser (AB Applied Biosystems) and fragment sizes were determined automatically using the GeneScan 3.1 Analysis software. Alleles were designated according to the number of trinucleotide repeats [13].

Molecular identification of non-*albicans* isolates was performed by sequencing the internal transcribed spacer (ITS) regions of ribosomal RNA genes. Sequence analysis was carried out using primers ITS1 (5ʹ TCCGTAGGTGAACCTGCGG 3ʹ) and ITS4 (5ʹ TCCTCCGCTTATTGATATGC 3ʹ), according to [16], and PCR products purified using the commercial Kit GenElute® PCR Clean-up (SIGMA). Sequences were edited with the Sequencer version 4.9 software package (Genes Codes Corporation), aligned with MEGA-X software [17] and compared by BLAST with sequences available from NCBI GenBank and ISHAM-barcoding database.

### Statistical *a*nalyses

Comparisons between categorical variables were performed using the Chi-square statistical test with Yates correction to compare between different conditions. Variables were compared using a 2-tailed t-test in order to evaluate significance associated with higher or lower CFUs values. A p value <0.05 was considered as significant. To evaluate if the statistically significant difference was correlated with helpful information in decision-making, the Cohen´s d value was calculated [18]. A d value below 0.1 indicates trivial effect, a value between 0.1 and 0.3 indicates a small effect, between 0.3 and 0.5 a moderate effect, and a value higher than 0.5 indicates a large difference effect.

The *C. albicans* CAI genotypes were compared according to their Shriver´s genetic distance, using PowerMarker v3.25 software [19]. A distance tree was constructed applying clustering with the unweighted pair group method with arithmetic means, using the MEGA-X software [17]. Comparison between groups was tested with the log-likelihood (G) statistic, using CAI genotypes [20]. Markov chain (MC) algorithm (1,000 iterations) was used to estimate, without bias, the exact p-value of this test [21]. These calculations were performed with Genepop sotware, version 4.2 [22].

## Results

### Yeast colonization in the oral cavity of healthy individuals

**Figure 1.**
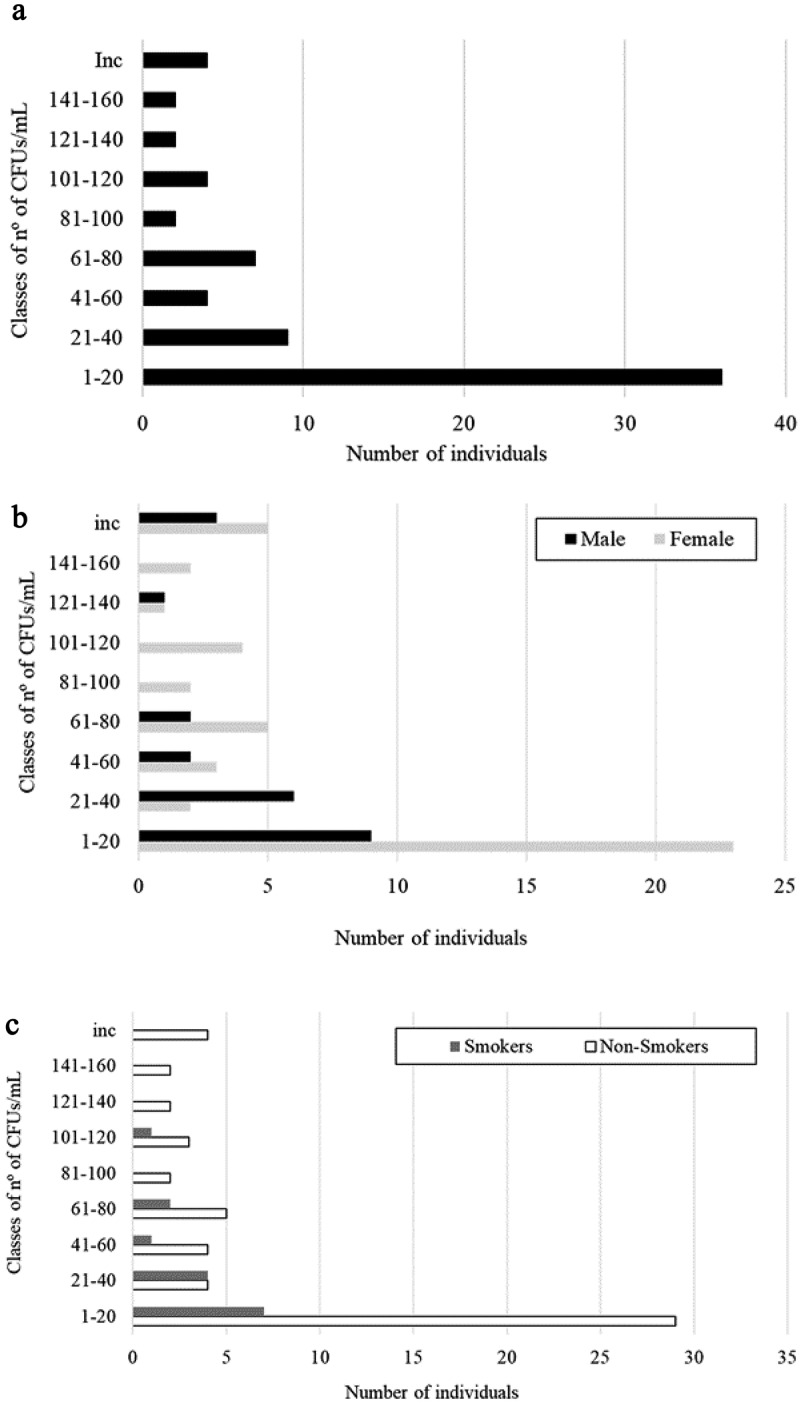
Distribution of the total individuals (A), males and females (B), and smokers and non-smokers (C) according to classes of yeast CFUs/mL in the saliva.

As previously reported, 181 healthy donors participated in this study, including students and staff of the University of Minho, among which 29 were couples. Results showed that 39% (70 out of 181) of the individuals carried yeasts in the oral cavity. The number of CFUs found in saliva samples was variable, with eight individuals showing a countless number of colonies (>300 CFUs) in the saliva, while others presented only one or two colonies. The majority of the individuals (55.7%) presented between one to 20 colonies (Figure 1A).

From the population studied, 116 were females and 65 were males. Although the number of males and females per classes of CFUs was different (Figure 1B), the percentage of colonized individuals did not differ significantly according to gender (40.5% of females and 35.4% of males were carriers), nor the level of colonization (number of CFUs) (not significant; p < 0.05). For comparative purposes, individuals with countless number of colonies were assigned as having 300 CFUs.

Eighteen percent (n = 34) of the participants were smokers. When correlating colonization with smoking habits (Figure 1C), the results showed that the number of colonized non-smokers was higher (n = 55) than colonized smokers (n = 15), but the differences were not statistically significant (not significant for p < 0.05).

To assess whether the intimacy results in cross-transmission of microorganisms, 29 couples were analysed. Interestingly, 11 couples (37.9%) showed no yeast colonization, nine (31.0%) presented colonization in only one member (five males and four females), and nine (31.0%) in both members of the couples.

Results were particularly significant when comparing colonized and non-colonized individuals between the couples and the remaining population (Yates chi-square is 16.09, p = 0.00006). Also, in the couples´ group, the average CFU number was 31.7, while in the remaining single individuals it was 13.9 CFUs (t-value is 2.15, p = 0.03, significant at p < 0.05, Cohen´s d = 0.36). CFU counts were also higher in the couples´ group with smoking habits (CFUs average = 26.3) than in single individuals (CFUs average = 6.1) with smoking habits (t-value is 2.23, p = 0.03, significant at p < 0.05, Cohen´s d = 1.23), contrary to CFUs number in non-smokers between the two groups (t-value is 1.73, p = 0.08, not significant at p < 0.05). These results indicate that the probability of colonization, together with high amount of yeast cells in the oral cavity, is moderately higher within the couples, but is largely potentiated by smoking habits. Colonization was likely not associated with hygiene habits or diet, because all the participants declared to follow regular oral hygiene habits and balanced diet.

### Identification of yeasts isolated from the oral cavity of healthy individuals

Differentiation of yeast isolates was performed on CHROMagar Candida medium. In this medium, *C. albicans* isolates formed yellow-green to blue-green colonies, while the remaining species presented a wide range of colours, from white to dark purple. However, since the colours may differ according to the strain, sequencing of the ribosomal internal transcribed spacer region was performed to identify non-*albicans* isolates, while *C. albicans* isolates were amplified with a CAI species-specific CAI marker [13]. The results confirmed that from the 70 samples positive for yeast species, 84.2% (n = 59) presented only *C. albicans* alone, 5.7% (n = 4) a mixed culture of *C. albicans* and other yeast species, 5.7% (n = 4) non-*albicans* species only, 2.8% (n = 2) *C. albicans* and bacteria, and in only one individual (1.4%) *C. albicans* co-inhabited the oral cavity with other yeast species and bacteria. Regardless of the presence of chloramphenicol in the culture medium, bacteria isolates were identified in five individuals, and from those, three also presented yeast colonization.

*Candida guilliermondi, C. lusitaniae, C. parapsilosis*, and *Rhodotorula mucilaginosa* were among the other yeast species identified (Table 1). The results also revealed that only one individual was colonized with *R. mucilaginosa* instead of *Candida* spp. None of the volunteers were colonized with more than two yeast species.

**Table 1.** Number and percentage of individuals with different colonization patterns in the oral cavity of the study subjects.

Patterns of yeast colonization	n (%)	Yeast species identified
Only *C. albicans*	59 (84.2)	*C. albicans*
*C. albicans* and other yeast species	4 (5.7)	*C.guilliermondii, C. lusitaniae*
Only non-*albicans* species	4 (5.7)	*Rhodotorula mucilaginosa, C. parapsilosis, C.guilliermondii*
*C. albicans* and bacteria	2(2.8)	*C. albicans*
*C. albicans*, other yeast species and bacteria	1 (1.4)	*C. albicans, C. lusitaniae*
**Yeast species identified**	**n (%)**	
*C. albicans*	66 (94.3)	
*C. guilliermondii*	4 (5.7)	
*C. parapsilosis*	2 (2.8)	
*C. lusitaniae*	2 (2.8)	
*R. mucilaginosa*	1 (1.4)	

### Genotyping of *C. albicans* isolates using CAI microsatellite

**Figure 2.**
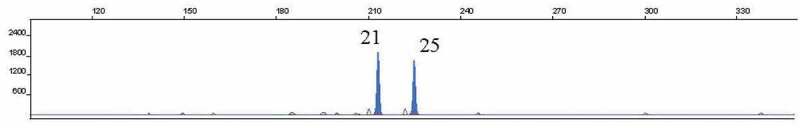
GeneScan profile of the most frequent CAI genotype (21–25). Electropherogram of PCR products obtained from a strain isolated from participant S019, showing allele 21 (213 base pairs) and allele 25 (225 base pairs).

As indicated above, 66 individuals were colonized with *C. albicans* strains which were genotyped using the CAI microsatellite marker. To identify different clones within the sample, a maximum of eight different colonies were genotyped. Results showed that a total of 39 distinct *C. albicans* genotypes were identified in isolates from the oral cavity of the 66 *C. albicans* colonized volunteers. From the 39 different genotypes identified, 13 were shared by more than one individual. The most frequent genotype found was 21–25 (Figure 2), present in 11 participants, followed by the genotype 25–25, shared by eight individuals (Supplementary data).

Fifty-three participants (80%) were colonized by strains with only one CAI genotype and 13 (20%) with two genotypes. The different genotypes isolated from the same individual were probably due to rearrangements at the CAI locus, such as slippage (in seven individuals) or loss of heterozygosity (LOH, in five individuals). The slippage is observed when one or two microsatellite repeated units are added or reduced in one or both alleles, as observed in strains isolated from individual S148, while the LOH is observed when one of the alleles is missing, as shown in strains isolated from the individual S031 (Table 2). Considering the internal structure of this microsatellite, a block of 10 repeat units distinguished alleles of group II (16 to 29) from group III (>30) [23]. This transition followed by rearrangements could be observed in strains isolated from the volunteer S142 between alleles 27 (group II) and 38 (Group III) (Table 2). In couples sharing *C. albicans* colonization, 55.6% (S035, S046, S071, S095 and S128) showed isolates with the same CAI genotype, 33.3% (S017, S036 and S054) presented isolates with different CAI genotypes, and only one couple (11.1%, S010) presented a mix of genotypes derived from slippage events, such as microvariations (Table 3).

**Table 2.** Individuals with different *C. albicans* CAI genotypes.

Individual	CAI Genotypes
S009	28–37 28–38
S013	25–25 26–26
S031	25–25 14-25
S032	27–54 27–27
S075	26–34 27–35
S078	25–25 26–26
S108	24–25 25–25
S140	24–26 24–24
S142	27–38 25–27
S148	21–26 21–28

**Table 3.** *Candida albicans* CAI genotypes and species identified in the couples.

Individual	CAI Genotypes/ Species
S001a	25–33
S001b	nc
S005a	25–25
S005b	nc
S010-a	19–26 18–25
S010-b	18–26 18–25
S015-a	*C. parapsilosis*
S015-b	26-26 *C. lusitaniae*
S013	25–25 26-26
S017-a	21–21
S017-b	23–27 24-28
S035-a	18–25
S035-b	18-25
S036-a	18-25
S036-b	25–32
S045-a	25–25
S045-b	nc
S046-a	21–26
S046-b	21–26
S050-a	nc
S050-b	18–29
S052-a	25-25
S052-a	nc
S054-a	26–34 *C. guilliermondii*
S054-b	21–21 *C. guilliermondii*
S070-b	nc
S070-b	28-38 *C. guilliermondii*
S071-a	21–25
S071-b	21–25
S073-a	nc
S073-b	21–25
S094-a	nc
S094-b	22–22
S095-a	12–20 *C. lusitaniae*
S095-b	12–20
S128-a	21–25
S128-b	21–25

nc: not colonized.

No significant difference was observed in CAI genotypes or alleles between couples in which both members were colonized, and couples in which only one member was colonized (p > 0.05). Interestingly, in couples colonized with species other than *C. albicans*, only the S054 couple also shared the non-*albicans* species, *C. guillermondii*. Couples S015 and S095 did not share the non-*albicans* species, *C. parapsilosis* or *C. lusitaniae* (Table 3).

**Figure 3.**
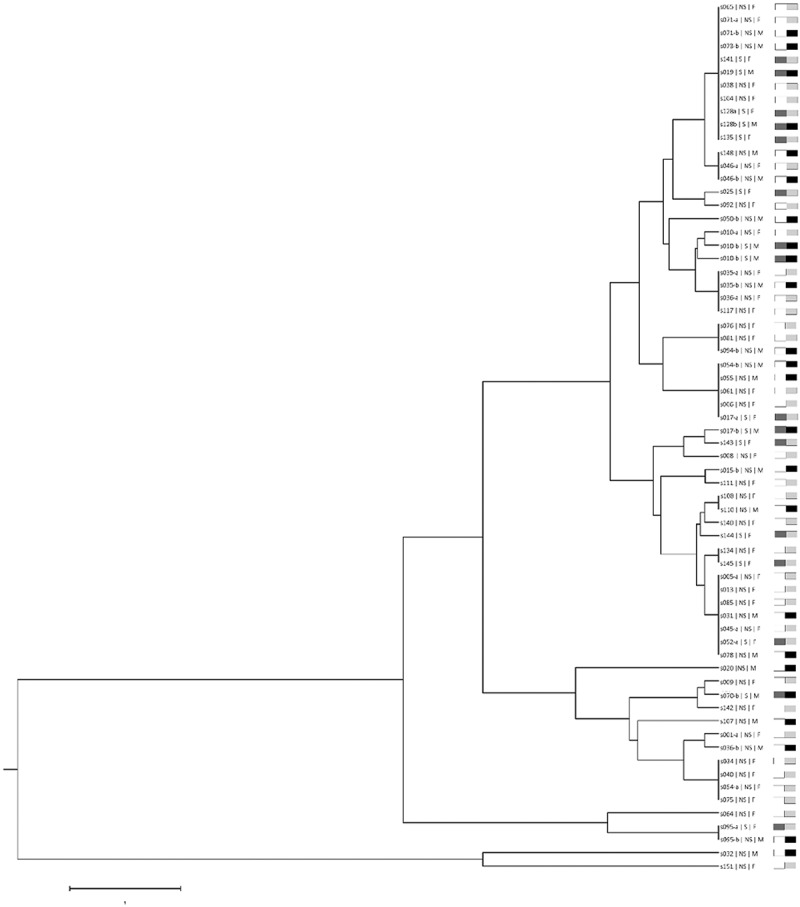
Dendrogram clustering *C. albicans* isolates according to their CAI genotype. S: Smoker; NS: non-smoker, F: Female, M: Male. Smokers/Male; Smokers/Female; Non-smokers/Male; Non-smokers/Female.

Figure 3 shows a dendogram correlating all CAI genotypes. For the dendrogram construction, in case of more than one CAI genotype present, only the most frequent was used. Results showed that there is no correlation between CAI genotypes and gender or smoking habits (p > 0.05).

### Stability of *C. albicans* genotypes over time

To evaluate whether *C. albicans* colonization is stable over time, 10 *C. albicans* carriers were selected for a second sampling, approximately 1 year after the first analysis. The collected isolates were again identified and *C. albicans* CAI genotypes compared with the first sampling. Results showed that the CAI genotypes of the colonizing strains were stable over 1 year (Table 4). Indeed, in seven individuals, the CAI genotype was the same as in the first analysis and, in subject S032, only one of the genotypes identified in the first analysis persisted after 1 year. Curiously, only individuals of the couple S054, lost yeast colonization after 1 year.

**Table 4.** Comparison of the genotypes found in the first and in the second sampling performed approximately one year later.

	1^st^ Sampling	2^nd^ Sampling
Individual	CAI Genotypes	CAI Genotypes
S001	(25–33)	(25–33)
S032	(27–54) (27–27)	(27–54)
S035-a	(18–25)	(18–25)
S035-b	(18–25)	(18–25)
S036-a	(18–25)	(18–25)
S036-b	(25–32)	(25–32)
S054-a	(27–34)	-
S054-b	(22–22)	-
S095-a	(12–20)	(12–20)
S095-b	(12–20)	(12–20)

## Discussion

The impact of yeast colonization on human health and disease is well established [9,24]. In this study, culture-dependent identification methods were used to evaluate oral yeast colonization in healthy individuals. Thirty-nine percent of the volunteers were colonized by yeasts, a number within the broad range reported in the literature: from 18.5% [25] to 92.5% [5]. This large range may be due to diverse potential confounding factors, including age, ethnicity, presence of neglected co-infection, as well as microbiological culture variables, including type of sample collected, culture media, time or temperature of incubation.

As observed in other studies [5,25], gender and oral hygiene habits (tooth brushing frequency) did not affect the percentage of yeast carriage or CFU counts. However, [26], in a crowdsourced population study reported that youth oral microbiomes are affected by gender and adult oral microbiomes by oral health habits (flossing frequency).

The relationship between smoking habits and oral yeast colonization has been a matter of debate. Some studies have shown that oral *Candida* prevalence is higher in smokers than in non-smokers [7], while other recent reports were inconclusive [27]. In the present work, smoking did not appear to increase yeast oral colonization or CFU counts in single volunteers, however, higher colonization and CFU counts were correlated with smoking in couples. The transferring of microorganisms through kissing, together with the described immunosuppressive nature of tobacco [28], may have contributed to this correlation. However, selection mechanisms resulting from a shared lifestyle, environment, or genetic factors from the host could also contribute. Three out of four participants with countless number of colonies (>300 CFUs) were within the couple non-smoking group.

In the present study, we observed that *C. albicans* was by far the most prevalent species (94.2%) among yeast carriers, which is also widely reported in the literature [29–32]. Indeed, in 84.2% of the volunteers, *C. albicans* was isolated alone and in 10% in a mixed culture, including chloramphenicol resistant bacteria. Curiously, *C. albicans* was identified together with *C. guillermondii* and *C. lusitaniae*, but not with *C. parapsilosis* or *R. mucilaginosa*. A lower competitiveness between *C. guillermondii* and *C. lusitaniae* with *C. albicans* may account for this result. *Candida tropicalis* and *C. glabrata* are frequently identified in oral samples, however, in our survey, these species were not found. Indeed, it is described that *C. glabrata* is innately resistant to antifungal agents [33], so the selection of volunteers that were not being treated with any antimicrobial compound may explain this result. The composition of the studied population (mostly young healthy adults) may also have influence on the oral population because functions of the innate immune system are downregulated in aged healthy volunteers compared with healthy young volunteers [34].

Another species frequently identified in oral samples is *C. dubliniensis*, but this species was also not isolated in our study. However, to the best of our knowledge, *C. dubliniensis* has not been identified yet in oral samples in Portugal [35,36].

Since the mode of reproduction of *C. albicans* is essentially clonal, it is described that variations in biological properties may be traced by only one variable marker [37]. However, due to evidence that some recombination may also occur in *C. albicans*, analysis of loci associated with known biological properties would provide more accurate data [37,38]. CAI microsatellite fulfils these criteria since it is located within the coding region of an important transcription factor, *RLM1* [23], and has been linked to some known biological properties [23,39,40], thus, justifying the use of only one marker. Genotyping of isolates from the 66 *C. albicans* colonized volunteers with the CAI marker identified a total of 22 different alleles and 39 distinct genotypes. Different studies have correlated strains presenting CAI alleles with 30 or more CAA/CAG repetitions with resistance to stress agents, virulence in the mouse model, and severity of human vulvovaginal candidiasis [23,39,40]. However, in this study no correlation was observed between strains with those CAI alleles and CFU counts. Similarly, no correlation was observed between any CAI genotypes and smoking habits or gender, possibly because only healthy participants were involved in the present study.

Results showed that the majority of the volunteers were colonized with the same CAI genotype, but some were colonized by two distinct genotypes that could result from a microevolutionary step, such as an LOH or slippage event [41]. This is a curious observation since this microsatellite is so polymorphic [13], yet at the oral surface of healthy participants, the local variability is, apparently, very reduced.

In hospitalized patients without oral infections, up to nine different *C. albicans* genotypes in the same individual were identified by using two molecular markers (Hist3 and CAI) [42]. Analysing only the CAI marker in the profile of isolates from [42] study, 74% of those patients (n = 17) had only one CAI genotype in the different clones, while 22% of the patients presented two CAI genotypes (n = 5). In the five cases with two CAI genotypes, only one could be explained by LOH, a simple mutational step, while the other four cases consisted of different CAI genotypes.

The results of the present study corroborated these findings, suggesting that in healthy individuals, only one *C. albicans* CAI genotype or microvariations of the same CAI genotype is predominant in the oral cavity. However, the difference between the two studies may indicate that hospitalized patients are more immunocompromised, disturbing the host-fungus interaction, enabling different CAI clones to inhabit the oral cavity. In CIn immunocompromised individuals the host-microorganism relationship changes from commensal to opportunistic and may eventually lead to the appearance of oral diseases [4,43,44].

In the couples group, it was interesting to observe that 37.9% were not colonized, 31.0% showed colonization in only one member of the couple, and only 31% in both members of the couple, indicating that transmission of *C. albicans* strains between couples is not a dominant phenotype, but the intimacy among couples increased the probability of heavy cross-colonization. It has been reported that a massive transmission of microbes may occur through kissing [45], so, related individuals are likely to share the oral microbiome [26]. However, when couples share *C. albicans* strains, the same or similar CAI genotype is predominant, suggesting an exogenous origin followed by CAI stabilization due to constraint sharing of microorganisms.

One important observation of the present study was that *C. albicans* strains are stable over time in the oral cavity, despite its continuous exposure to the external environment. In all individuals the same CAI genotypes persisted after 1 year, except one case with two CAI genotypes in which only the CAI genotype with a high molecular weight allele persisted. In agreement with this, it has been described that high molecular weight CAI alleles could confer higher adaptability to certain niches, such as mucosal surfaces, enabling specific clones to persist [23,40,46], Carvalho-Pereira *et al*. 2018. Our results also indicate that the cross-colonization between the two members of the couple is potentiated when the mates are smokers.

Overall, our study presents interesting new findings regarding yeast colonization in healthy individuals and, as regards *C. albicans* strains, the stability of the present genotypes over time. Nevertheless, we acknowledge the existence of some limitations regarding the number of couples included in the study and the need for further information about the yeast isolates, for instance, their antifungal susceptibility profiles.

## Supplementary Material

Supplemental Material
